# Association between breastfeeding and eczema during childhood and adolescence: A cohort study

**DOI:** 10.1371/journal.pone.0185066

**Published:** 2017-09-25

**Authors:** Jingying Wang, Alban Ramette, Maja Jurca, Myrofora Goutaki, Caroline S. Beardsmore, Claudia E. Kuehni

**Affiliations:** 1 Institute of Social and Preventive Medicine, University of Bern, Bern, Switzerland; 2 Paediatric Respiratory Medicine, Children’s University Hospital of Bern, Bern, Switzerland; 3 Department of Infection, Immunity and Inflammation, University of Leicester, Leicester, United Kingdom; 4 Leicester Respiratory Biomedical Research Unit, University of Leicester, Leicester, United Kingdom; University Children`s Hospital Zurich, SWITZERLAND

## Abstract

**Background:**

Breastfeeding is said to protect children from eczema (atopic dermatitis), but the available evidence is conflicting and subject to the influences of parental atopy and reverse causation (when mothers extended duration of breastfeeding because their children had eczema).

**Methods:**

In the prospective, population-based Leicester Respiratory Cohort study, we assessed duration of breastfeeding in children aged 1–4 years. Prevalence of eczema was determined by questionnaire surveys that were repeated until the children were 17 years old. We investigated the association between having been breastfed and current eczema using generalized estimating equations, adjusting for potential confounders, and tested for effect modification by parental atopy. We also assessed the association between having been breastfed and incident eczema at ages 2, 4, and 6 years using multivariable logistic regression.

**Results:**

Among the 5,676 children in the study, 2,284 (40%) had never been breastfed, while 1,610 (28%), 705 (12%), and 1,077 (19%) had been breastfed for 0–3, 4–6, and >6 months, respectively. Prevalence of current eczema decreased from 36% in 1-year-olds to 18% in children aged 10–17 years. Breastfeeding was not associated with current eczema. Compared with children who had never been breastfed, the adjusted odds ratios for current eczema at any age were 1.02 (95% confidence interval 0.90–1.15) for children who had been breastfed for 0–3 months, 0.97 (0.82–1.13) for children breastfed for 4–6 months, and 0.98 (0.85–1.14) for children breastfed for >6 months. There was no strong evidence for an effect modification by parental atopy (p-value for interaction term was 0.061) and no association between having been breastfed and incident eczema later in childhood.

**Conclusions:**

This population-based cohort study found no evidence for protection of breastfeeding against childhood eczema at any age, from infancy through adolescence.

## Introduction

Eczema or atopic dermatitis is the most common chronic inflammatory skin disorder in children [1, 2]. Its prevalence has increased in recent decades and imposed a burden on affected children, their families, and the health care system [3–5]. Eczema is often the first manifestation of atopy, and is associated with concurrent and later food allergy, asthma, and allergic rhinitis—the so-called “atopic march” [6]. Thus, better knowledge of risk factors and preventive strategies for childhood eczema would be useful in public health [7].

As early as the 1930s, breastfeeding was reported to prevent development of childhood eczema [8]. Several early studies supported this [9–12]. More recent studies that were larger and better controlled for confounding have tended to find no evidence for a protective effect [13–16], or have even suggested that breastfeeding increased the risk for eczema [17–20]. However, some of these studies assessed eczema only during infancy or preschool ages [15, 18, 19], and only a few had long-term follow-up [12, 16, 20]. Parental atopy has been reported to modify the association [10, 19, 21], with a stronger protective effect of breastfeeding in children with a family history of atopy [10, 19, 21]. However, a systematic review of 21 cohort studies on breastfeeding and eczema found that only six had stratified analyses by parental atopy [14]. Finally, reverse causation can affect results when mothers of children with eczema breastfeed longer [22] than mothers of children without eczema, because they believe breast milk could help reduce or cure their baby’s rash.

In this large population-based cohort study, we determined if duration of breastfeeding was associated with prevalence of eczema from age 1 to 17 years, assessed a potential effect modification by parental atopy, and investigated the association with incident eczema to avoid reverse causation.

## Methods

### Study design and study population

We analysed data from the 1998 Leicester Respiratory Cohort, a prospective, population-based cohort study in Leicestershire, UK, described in detail elsewhere [23]. A random sample of 8,700 children, stratified by age and ethnicity (white and South Asian), was extracted from the local child health database. Perinatal data came from birth records, and data on immunisation and growth were collected from health visitor records. The Leicester 1998 Cohort consists of two subcohorts. One cohort, Cohort a, included 4,400 children born between May 1993 and April 1997, aged 1–4 years in 1998, and was primarily designed to enable comparison with a previous cohort of children, born eight years earlier (the 1990 Leicester Cohort). Cohort b included 4,300 children born between May 1996 and April 1997 who were 1 year old in 1998. While the general study methodology was similar, a few questionnaires differed slightly between the two subcohorts.

All parents received an initial postal questionnaire in 1998, when the children were 1–4 years old, which asked about atopic diseases, respiratory symptoms, and potential risk factors. These surveys were repeated in 2001, 2003, 2006, and 2010. Parents in Cohort b received an additional questionnaire in 1999. The response rate in 1998 was 78% (6,808/8,700), but lower afterwards.

The Leicestershire Health Authority Research Ethics Committee approved this study. Informed consent was obtained for all the children participating, from their parents or legal guardians.

### Definitions of breastfeeding and current eczema

The total duration of breastfeeding (regardless of exclusivity) was surveyed with the baseline questionnaire using the questions “Was your child breastfed? Yes/No”, and “If yes, for how long: 0–3 months, 4–6 months or >6 months.” Both questions on breastfeeding showed excellent repeatability when asked again three months later, with an unweighted Cohen’s kappa of 0.96 (95% CI 0.94–0.99) for the question on any breastfeeding (Yes/No) and an intraclass correlation coefficient (ICC) of 0.95 (95% CI 0.94–0.96) for the question on duration of breastfeeding [24].

We defined current eczema (any eczema) by using the following question derived from the International Study of Asthma and Allergies in Childhood (ISAAC) [13]:“In the past 12 months, has your child had eczema (an itchy rash on arms, face and legs)?” In Cohort b we asked parents this question in each questionnaire from 1998 to 2006, and asked children “Have you had eczema in the last 12 months?” in 2010. For children of Cohort a, the questions were only asked in surveys 2003, 2006, and 2010 (S1 Table).

### Statistical analyses

To assess the association between duration of breastfeeding and current eczema, we included data of all children whose parents replied to the baseline questionnaire survey and provided information on breastfeeding and current eczema.

Due to the range in birth years (1993–7), children were 1–4 years old at the first survey in 1998 and 13–17 in 2010 (S1 Fig). We divided data into seven age groups: 1-year-olds, 2-year-olds, 3–4-year-olds, 5–6, 7–9, 10–13, and 14–17-year-olds (S1 Fig). Children were included only once in each age group, but could have contributed observations from different surveys to several age groups. Data were analysed by age groups, rather than by the calendar year of the surveys.

We evaluated the association between duration of breastfeeding and current eczema during childhood and adolescence by using generalized estimating equations (GEE) [25], and accounting for the correlated responses of each child over time [26]. We calculated crude and adjusted associations through the GEE approach. We considered the following variables as potential confounders because they have been reported to be associated with both breastfeeding and eczema (S2 Fig): age [13, 17], sex [13, 17, 19], ethnicity [27–29], family education [15, 17, 19, 30], Townsend deprivation index (an area-based deprivation score) [31], day care attendance [14, 15, 19], number of older siblings [14, 17, 19, 30], maternal pre- and postnatal smoking [17, 19, 30], pet ownership (cats, dogs, or birds) [15, 19, 32], and parental atopy (paternal or maternal history of asthma, hay fever, or eczema) [10, 13, 15, 17, 19, 21, 30].

We tested for effect modification by first including interaction term (duration of breastfeeding and parental atopy) in the adjusted GEE model and then stratifying the adjusted GEE model by parental atopy.

We ruled out reverse causation by performing separate analyses in which we included only children of Cohort b, and excluded those who had current eczema at the baseline survey. We determined whether duration of breastfeeding was associated with incident eczema (eczema developed in children who had no eczema at the age of 1 year) at ages 2 (survey in year 1999), 4 (survey in year 2001), and 6 (survey in year 2003) by using logistic regression, models were adjusted for all the aforementioned confounders, except for age.

We performed three sensitivity analyses to test the robustness of our findings. In the first, we assessed the association between duration of breastfeeding and current eczema for each of the seven age groups separately using logistic regression adjusting for all the mentioned confounders. In the second, we tested for effect modification specifically by maternal atopy, sex, ethnicity and maternal smoking, repeating all analyses. In the third, we redefined the outcome as eczema reported at least twice throughout the follow up to avoid our results being influenced by a large number of children who had only had one episode of eczema, i.e. very mild eczema. For this sensitivity analysis, we included only children of Cohort b who had participated in at least two surveys.

All the data were prepared and analysed using Stata (Version 14.1, Stata Corporation, Austin, Texas, USA).

## Results

In total, 5,676 children answered to the baseline survey and at least one of the questionnaires asking about eczema (S3 Fig). Among these children, 2,319 (41%) came from Cohort a, and 3,357 (59%) came from Cohort b (S3 Fig). In total, 2,943 (52%) were male, 4,333 (76%) were white, and 1,343 (24%) were of South Asian ethnic origin. Forty percent (2,284) had never been breastfed, while 1,610 (28%) had been breastfed for 0–3 months, 705 (12%) were breastfed for 4–6 months, and 1,077 (19%) were breastfed for >6 months (Table 1). Among the 3,357 children of Cohort b, 1,191 (35%) reported current eczema at the age of 1 year in 1998, and 2,135 (64%) did not (S3 Fig).

**Table 1:** Characteristics of the study population at the age of 1–4 years in 1998 (N = 5,676).

		n	(%)
**Demographic factors**			
Age groups (in years)	1	3,788	67
	2	683	12
	3	596	10
	4	609	11
Gender	Female	2,733	48
	Male	2,943	52
Ethnicity	Whites	4,333	76
	South Asian	1,343	24
**Breastfeeding**	Never	2,284	40
	0–3 months	1,610	28
	4–6 months	705	12
	>6 months	1,077	19
**Socioeconomic status**			
Townsend deprivation index^**a**^	More affluent	1,307	23
	Affluent	1,266	22
	Average	1,170	21
	Deprived	995	17
	More deprived	846	15
Family education^**b**^	High	2,426	43
**Environmental exposures**			
Day care attendance		2,330	41
Number of older siblings	0	2,347	41
	1 or 2	2,895	51
	> = 3	424	7
Mother smoking during pregnancy		865	15
Mother currently smoking		1,115	20
Pet (dog/cat/bird) ownership		2,174	38
**Parental history of atopy (asthma, eczema, or hay fever)**			
Maternal atopy		2,519	44
Paternal atopy		2,108	37
**Parental history of eczema**			
Maternal eczema		1,270	22
Paternal eczema		707	12

^a^ An area-based deprivation score; higher values indicate greater deprivation.

^b^ Parents who completed their full-time education at the age of 16 or older.

Children excluded from the analysis (N = 1,035) were more of South Asian ethnic origin (p<0.001), and less breastfed (p = 0.002) than included children (S2 Table). More excluded children had deprived socioeconomic background (p<0.001), and parents with low education level (p = 0.035) than included children. Furthermore, significantly more excluded children attended day care (p<0.001), and had mothers smoked during pregnancy (p = 0.001) and less of them had pets at home (p = <0.001) (S2 Table).

### Association between breastfeeding duration and prevalence of current eczema during childhood and adolescence

**Fig 1:**
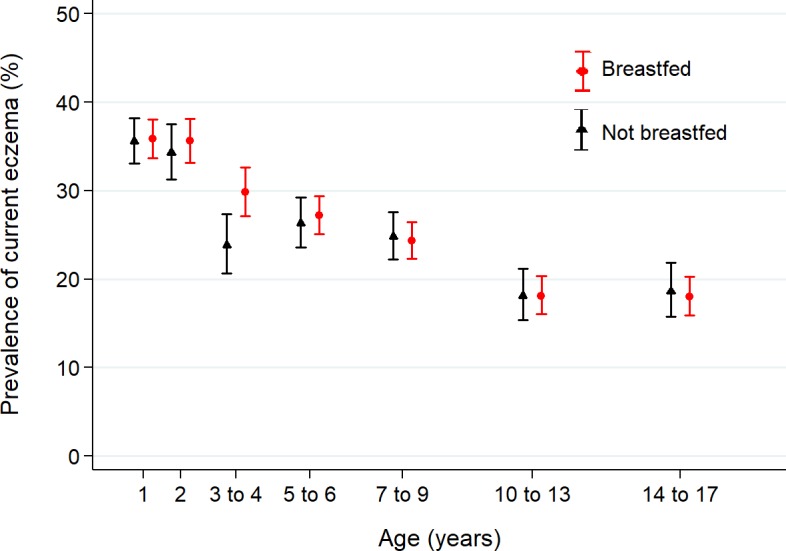
Prevalence of current eczema during childhood and adolescence, by breastfeeding status.

Prevalence of current eczema was 36% in 1-year-olds, 35% in 2-year-olds, 28% at age 3–4, 27% at age 5–6, 25% at age 7–9, and 18% at ages 10–13 and 14–17 years, and did not differ between those who had been breastfed and those who had not (Fig 1, S3 Table).

Duration of breastfeeding was also not associated with prevalence of current eczema during childhood and adolescence. In the unadjusted GEE model, the odds ratios (ORs) were 1.03 (0.91–1.16) for children who had been breastfed for 0–3 months, 1.02 (0.87–1.18) for those breastfed 4–6 months, and 0.98 (0.86–1.12) for those breastfed longer than 6 months. In the adjusted GEE model, respective ORs were 1.02 (0.90–1.15), 0.97 (0.82–1.13), and 0.98 (0.85–1.14) for breastfeeding durations of 0–3, 4–6, and over 6 months (Table 2). There was no strong evidence for an effect modification by parental atopy (p-value of interaction term was 0.061), and stratification by parental atopy (Table 2) did not affect findings.

**Table 2:** Association between breastfeeding duration and prevalence of current eczema during childhood and adolescence.

**Overall association between breastfeeding and current eczema**				
	**Unadjusted model (N = 5,676)**		**Adjusted**^**a**^ **model (N = 5,456)**	
Breastfeeding duration	OR (95% CI)	p-value	OR (95% CI)	p-value
No breastfeeding	1.00	-	1.00	-
0–3 months	1.03 (0.91–1.16)	0.641	1.02 (0.90–1.15)	0.802
4–6 months	1.02 (0.87–1.18)	0.846	0.97 (0.82–1.13)	0.668
>6 months	0.98 (0.86–1.12)	0.785	0.98 (0.85–1.14)	0.827
**Association between breastfeeding and current eczema modified by parental atopy**				
	**Parents with history of atopy (n = 3,447)**		**Parents without history of atopy (n = 2,009)**	
Breastfeeding duration	OR (95% CI)	p-value	OR (95% CI)	p-value
No breastfeeding	1.00	-	1.00	-
0–3 months	0.98 (0.84–1.14)	0.776	1.07 (0.85–1.34)	0.582
4–6 months	1.08 (0.90–1.31)	0.408	0.76 (0.56–1.04)	0.085
>6 months	1.06 (0.90–1.26)	0.476	0.89 (0.69–1.15)	0.362

Data are presented as odds ratios (ORs) with their 95% confidence intervals (CIs) and associated p-values, both for unadjusted and adjusted GEE models.

The baseline group consisted of children who had not been breastfed.

Adjusted models include age, sex, ethnicity, family education, Townsend deprivation index, day care attendance, number of older siblings, pet ownership (dog, cat, or bird), pre- and postnatal maternal smoking.

^a^ Adjusted for all factors listed above, plus parental atopy (defined as paternal or maternal history of asthma, hay fever, or eczema).

### Association between breastfeeding duration and subsequent incident eczema

To avoid a potential systematic bias by reverse causation, we analysed subsequent incident eczema in the 2,135 children from Cohort b who had no eczema at the age of 1 year (S3 Fig). Among the 1,409 who participated at the age of 2 years in the 1999 survey, 236 (17%) reported current eczema, and this did not differ by duration of breastfeeding (Table 3). The same was true for current eczema reported at the ages of 4 (15%, 198 out of 1,347) or 6 years (16%, 191 out of 1,139) (Table 3).

**Table 3:** Associations between breastfeeding duration and incident eczema at the ages 2, 4, and 6 years.

**At age 2 years (1999 survey)**				
	**Unadjusted (n = 1,409)**		**Adjusted**^a^ **(n = 1,373)**	
Breastfeeding duration	OR (95% CI)	p-value	OR (95% CI)	p-value
No breastfeeding	1.00	-	1.00	-
0–3 months	1.04 (0.73–1.48)	0.844	1.14 (0.78–1.66)	0.487
4–6 months	1.15 (0.74–1.80)	0.534	1.16 (0.72–1.87)	0.542
>6 months	1.28 (0.88–1.85)	0.195	1.48 (0.99–2.21)	0.053
**At age 4 years (2001 survey)**				
	**Unadjusted (n = 1,347)**		**Adjusted**^a^ **(n = 1,315)**	
Breastfeeding duration	OR (95% CI)	p-value	OR (95% CI)	p-value
No breastfeeding	1.00	-	1.00	-
0–3 months	1.06 (0.72–1.56)	0.763	1.01 (0.67–1.52)	0.947
4–6 months	1.31 (0.82–2.09)	0.257	1.29 (0.79–2.12)	0.305
>6 months	1.26 (0.84–1.91)	0.261	1.24 (0.80–1.93)	0.343
**At age 6 years (2003 survey)**				
	**Unadjusted (n = 1,139)**		**Adjusted**^a^ **(n = 1,104)**	
Breastfeeding duration	OR (95% CI)	p-value	OR (95% CI)	p-value
No breastfeeding	1.00	-	1.00	-
0–3 months	0.98 (0.67–1.43)	0.904	0.96 (0.64–1.43)	0.826
4–6 months	0.81 (0.48–1.37)	0.429	0.85 (0.49–1.48)	0.568
>6 months	0.93 (0.61–1.42)	0.746	0.84 (0.53–1.31)	0.440

Data are presented as odds ratios (ORs) with their 95% confidence intervals (CIs) and associated p-values, both in unadjusted and adjusted logistic regression models.

The baseline group consisted of children who had not been breastfed.

^a^ Adjusted for sex, ethnicity, family education, Townsend deprivation index, day care attendance, number of older siblings, pet ownership (dog, cat, or bird), pre- and postnatal maternal smoking, and parental atopy (defined as paternal or maternal history of asthma, hay fever, or eczema).

### Sensitivity analyses

When we re-examined the data by conducting adjusted logistic regressions for each age group (instead of merging outcomes at all ages in GEE analyses), we also found no consistent association between breastfeeding duration and current eczema (S5 Table). Stratification by maternal atopy, sex, ethnicity, or maternal smoking did not affect results of association between breastfeeding and current eczema (S6 Table).

Finally, we repeated all analyses for children suffering from eczema in at least two surveys, to define a clinically more relevant outcome. In total, 2,983 children of Cohort b participated in two or more surveys. Among these, 980 (33%) reported having eczema in the preceding 12 months in at least two surveys. Breastfeeding was not associated with children who reported eczema at least twice (S8 Table).

## Discussion

This large cohort study, which followed children up to the age of 17, found no evidence for associations between breastfeeding and prevalence or incidence of eczema throughout childhood (S4 Fig), and no evidence of effect modification by parental or maternal atopy.

Much, but not all previous work agrees with these findings. Early studies reported breastfeeding protects against eczema [9–11]. By examining more recent evidence, neither a review of Yang et al. that summarized 21 prospective cohort studies on breastfeeding and atopic dermatitis published between 1966 and 2008 [14], another review including 24 studies on breastfeeding and eczema [33], nor a Cochrane review summarizing findings for breastfeeding and different health outcomes [34] support earlier claims for breastfeeding. However, an analysis of data from the Tasmanian Asthma Cohort [16] with a follow-up from age 7 to age 44 (no other study has had such a long follow-up) reported a protective effect against infantile (baby) eczema in children with a maternal history of atopy. Both breastfeeding and infantile eczema were assessed retrospectively when children were 7 years old in this study, this might introduce great recall bias, and subsequently overestimated the effect of breastfeeding on eczema. Parental, and particularly maternal atopy also has been reported to modify the association between breastfeeding and eczema, with stronger protective effects in children with atopic parents [10, 19, 21]. But the review by Yang et al. did not report such an effect [14].

Our study may bring a measure of clarity to this equivocal literature (and perhaps inform conventional wisdom as well). The study derives methodological strength from its large size, as well as its having both controlled for numerous potential confounding factors and tested for effect modifications by parental, maternal atopy, sex, ethnicity and maternal smoking. We had sufficient sample size to detect an odds ratio of 0.85 with a power of 80%, this would, in our view be a relevant reduction in risk. However, we cannot of course exclude small reductions in the risk that our study might not have had the power to detect. We also had longitudinal data with 12 years of follow-up covering the entire period of childhood and adolescence. Most other studies have used logistic regression to analyse prevalence of eczema at specific ages [21, 30], which is a cross-sectional approach. A further methodological attribute of our study that potentially helps resolve the eczema and breastfeeding picture is its analysis of data with GEE. This method has been widely used in the longitudinal analysis of epidemiological data [35], but has only rarely been used to study relationship between breastfeeding and eczema [17, 20]. Finally, to rule out the possibility of reverse causation we also studied incident eczema in children who had been asymptomatic during infancy.

Among our study’s limitations, breastfeeding was assessed at the age of 1 year and relied on parental recall. One study has suggested that parents whose children had eczema were more likely than parents whose children did not have eczema to recall breastfeeding and its duration [13]. However, the breastfeeding question in our questionnaire showed excellent overall repeatability (Cohen’s kappa = 0.96) [24], which did not differ between children with and without eczema (Cohen’s kappa = 0.99 and 0.92 respectively) (S9 Table). Our questionnaires did not record breastfeeding exclusivity, nor the information on what and when the supplementary food was introduced. We also did not ask about the application of moisturizers during infancy, which was suggested as a method to prevent development of atopic dermatitis in children [36]. Some might also consider this study’s lack of clinical confirmation of the presence of eczema a limitation.

Our study provides no evidence of an association between breastfeeding and prevalence or incidence of eczema during childhood and adolescence, or an effect modification by parental atopy. While our study does not suggest a relevant protective effect against eczema, breastfeeding should further be strongly recommended because of its many nutritional, immunological, psychological, and economic benefits.

## Supporting information

S1 Fig Age range of children from Leicester Respiratory Cohorts who participated in each survey. Restricted to 5,676 children remaining in the analysis.
(PDF)

S2 Fig Directed acyclic graph (DAG). (PDF)

S3 Fig Flow diagram of study children. (PDF)

S4 Fig Summary of association between prolonged breastfeeding (>6 months) and eczema. (PDF)

S1 Table Age in 1998 and summary of eczema questions asked in different surveys. (PDF)

S2 Table Characteristics of participants and dropouts at baseline survey. (PDF)

S3 Table Prevalence of current eczema at different age groups by breastfeeding status. (PDF)

S4 Table Association between breastfeeding duration and prevalence of current eczema during childhood and adolescence—restricted to children with complete information on all the confounders. (PDF)

S5 Table Association between breastfeeding duration and current eczema at different age groups. (PDF)

S6 Table Association between breastfeeding duration and prevalence of current eczema during childhood and adolescence—Modified by maternal atopy, sex, ethnicity and maternal smoking. (PDF)

S7 Table Associations between breastfeeding duration and incident eczema at ages 2, 4, and 6 years—restricted to children with complete information on all the confounders. (PDF)

S8 Table Association between breastfeeding duration and recurrent eczema. (PDF)

S9 Table Level of agreement of responses for children with and without eczema who provided breastfeeding responses (yes/no) at baseline survey and in subsequent questionnaires. 3-month interval, 1-year-old children, n = 463.
(PDF)
