# Supplementary material for: Association between breastfeeding and eczema during childhood and adolescence: A cohort study
Source: PLoS One. 2017 Sep 25;12(9):e0185066. doi: 10.1371/journal.pone.0185066 (PMC5612686; doi:10.1371/journal.pone.0185066)

S1 Fig. Age range of children from Leicester Respiratory Cohorts who participated in each survey

(Restricted to 5,676 children remaining in the analysis)

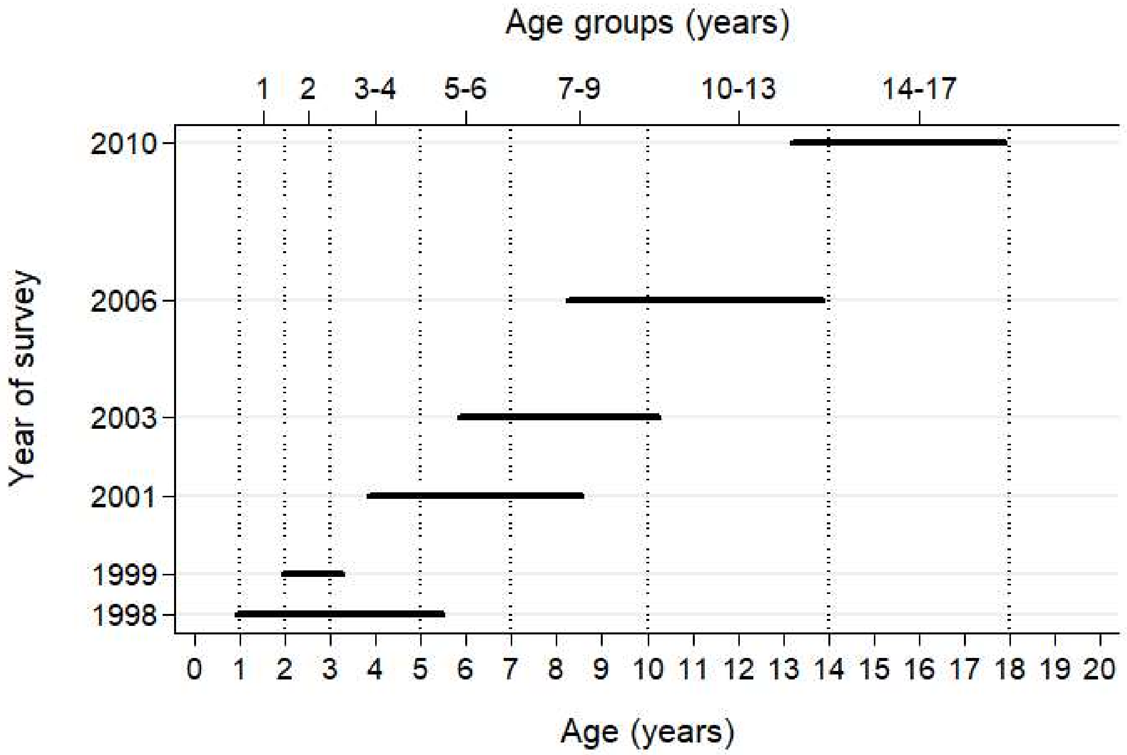

Supplement: S1 Fig — Restricted to 5,676 children remaining in the analysis. (PDF) [file pone.0185066.s001.pdf]
