# Supplementary figures and images for: Association between breastfeeding and eczema during childhood and adolescence: A cohort study
Source: PLoS One. 2017 Sep 25;12(9):e0185066. doi: 10.1371/journal.pone.0185066 (PMC5612686; doi:10.1371/journal.pone.0185066)

**S2 Fig. Directed acyclic graph (DAG)**

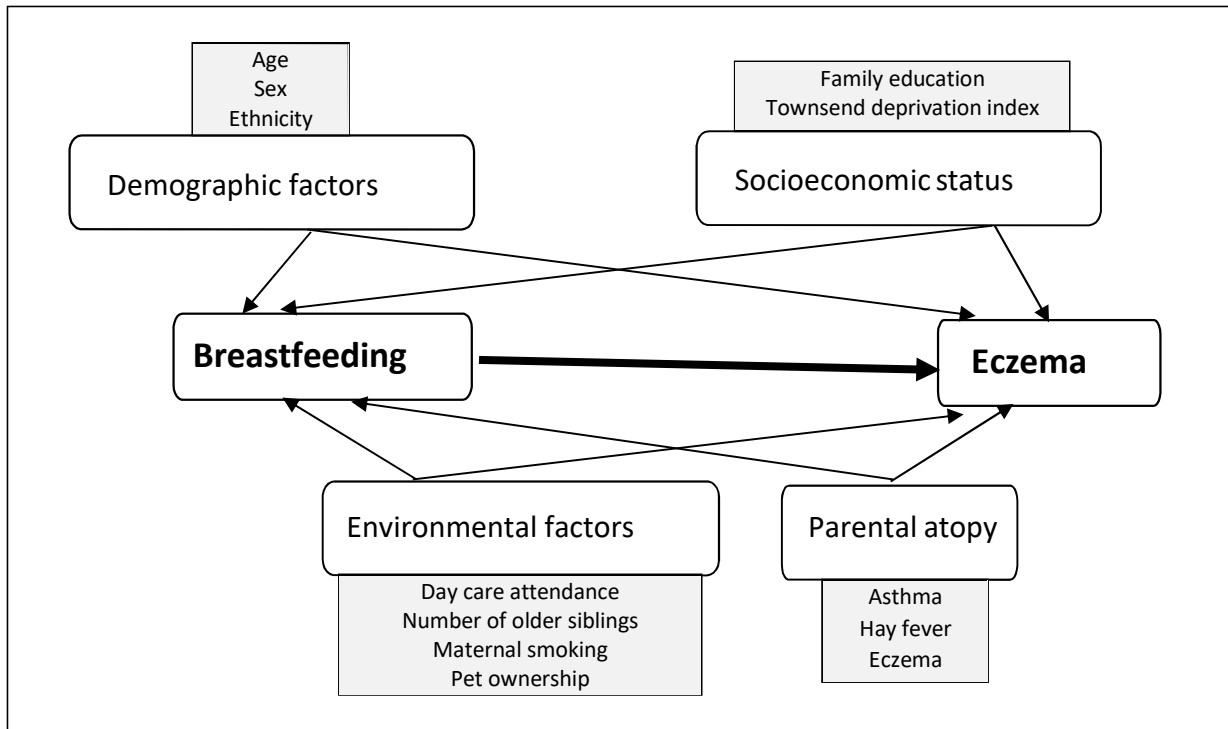

Supplement: S2 Fig — (PDF) [file pone.0185066.s002.pdf]

**S3 Fig. Flow diagram of study children**

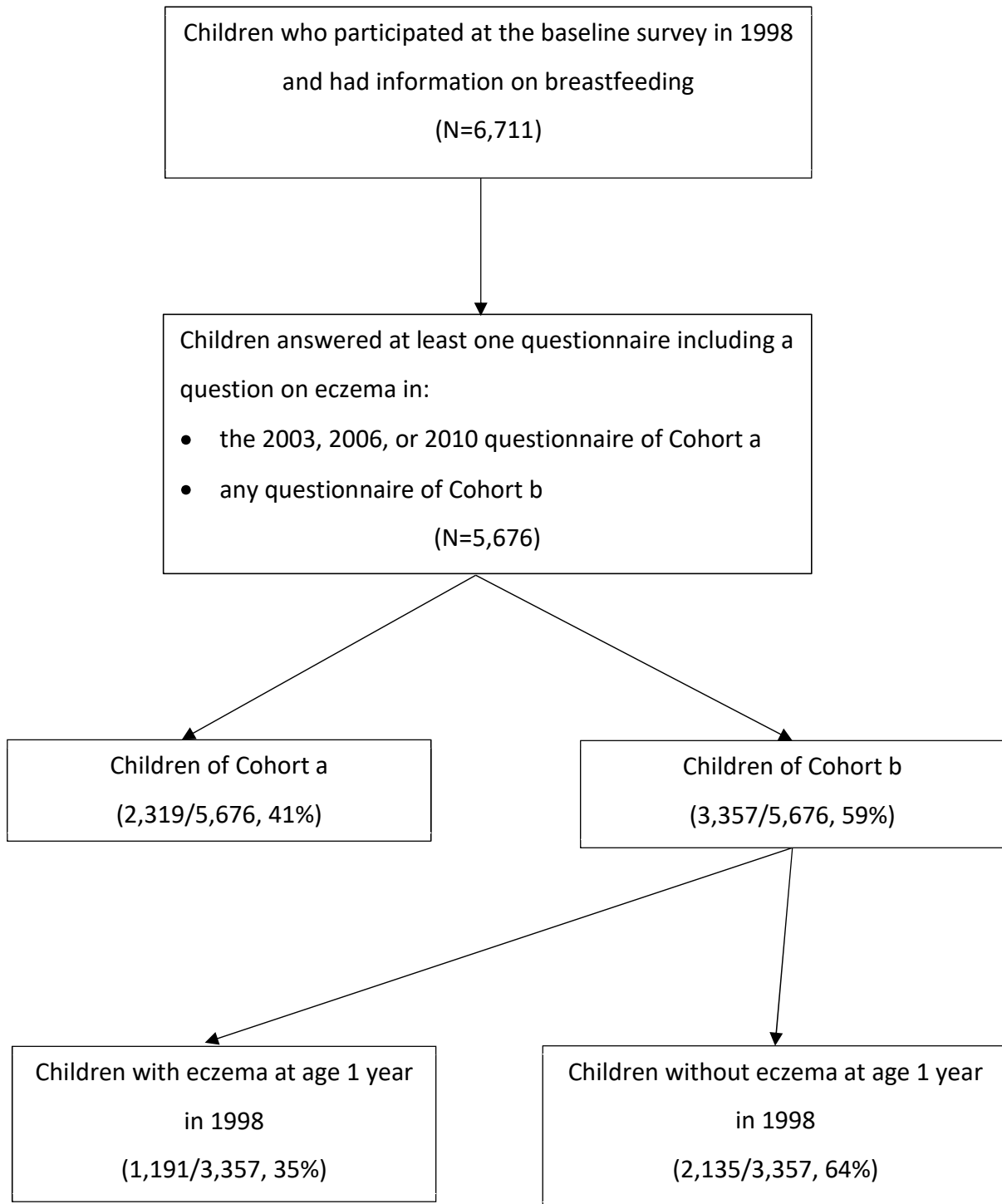

Supplement: S3 Fig — (PDF) [file pone.0185066.s003.pdf]
