# Supplementary material for: Association between breastfeeding and eczema during childhood and adolescence: A cohort study
Source: PLoS One. 2017 Sep 25;12(9):e0185066. doi: 10.1371/journal.pone.0185066 (PMC5612686; doi:10.1371/journal.pone.0185066)
Supplement: S4 Fig — (PDF) [file pone.0185066.s004.pdf]

S4 Fig. Summary of effects of prolonged breastfeeding (>6 months) on eczema

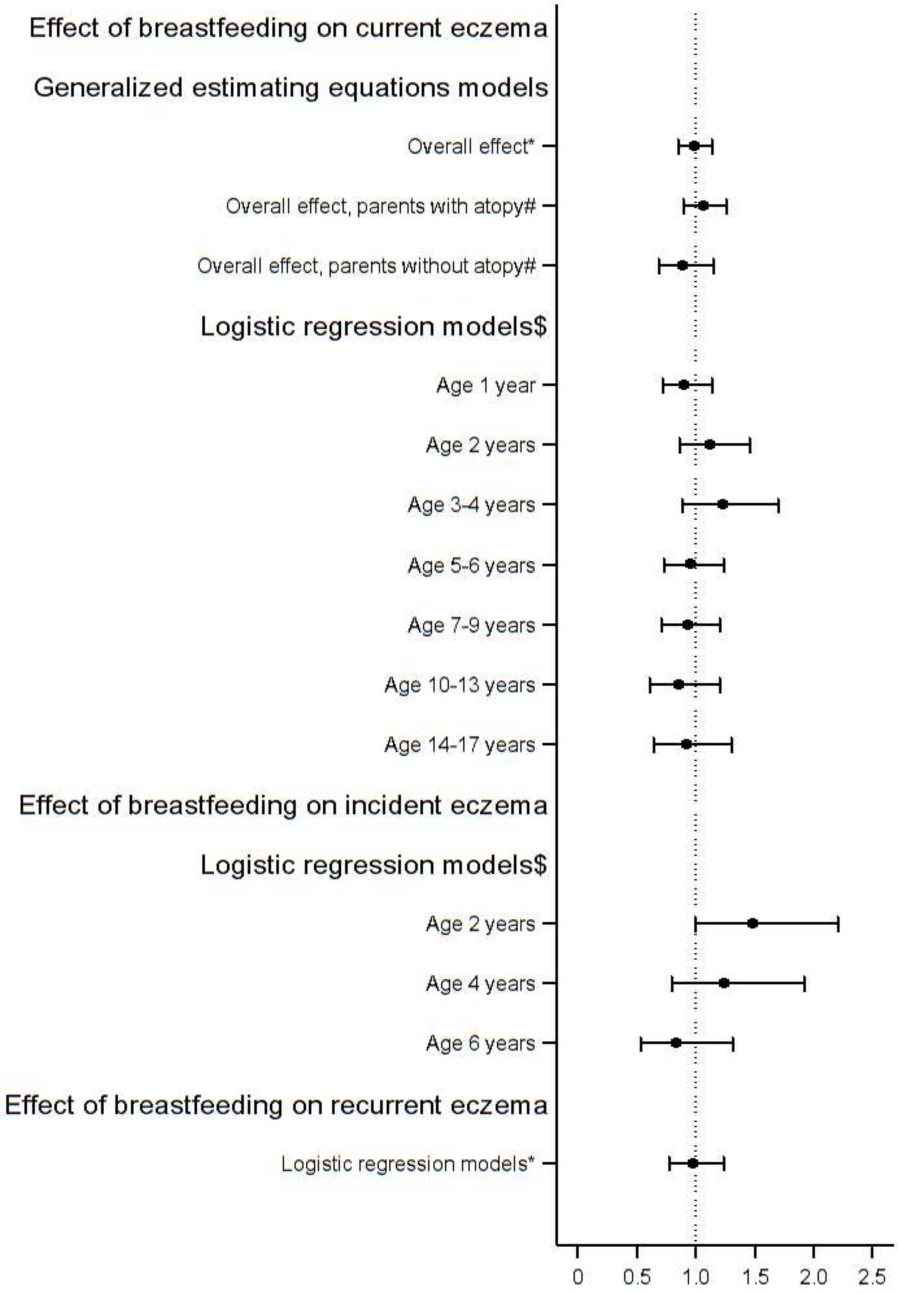

Data are presented as odds ratios (ORs) with their 95% confidence intervals (CIs).
The baseline group consisted of children who had not been breastfed.
All models were adjusted for sex, ethnicity, family education, Townsend deprivation index, day care attendance, number of older siblings, pet ownership (dog, cat, or bird), pre- and postnatal maternal smoking.
*Adjusted additionally for age and parental atopy (defined as paternal or maternal history of asthma, hay fever, or eczema).
Adjusted additionally for age.
$ Adjusted additionally for parental atopy.
