# Supplementary material for: Association between breastfeeding and eczema during childhood and adolescence: A cohort study
Source: PLoS One. 2017 Sep 25;12(9):e0185066. doi: 10.1371/journal.pone.0185066 (PMC5612686; doi:10.1371/journal.pone.0185066)
Supplement: S1 Table — (PDF) [file pone.0185066.s005.pdf]

S1 Table. Age in 1998 and summary of eczema questions asked in different surveys

	Participating cohorts	
	**Cohort a**	**Cohort b**
**Age in 1998**	1-4 years	1 year
**Surveys**	**Questions on eczema**	
1998	No question on eczema	In the past 12 months, has your child had eczema?
1999	No survey	In the past 12 months, has your child had eczema?
2001	No question on eczema	In the past 12 months, has your child had eczema?
2003	In the past 12 months, has your child had eczema?	In the past 12 months, has your child had eczema?
2006	In the past 12 months, has your child had eczema?	In the past 12 months, has your child had eczema?
2010	Have you had eczema in the last 12 months?	Have you had eczema in the last 12 months?
