# Supplementary material for: Association between breastfeeding and eczema during childhood and adolescence: A cohort study
Source: PLoS One. 2017 Sep 25;12(9):e0185066. doi: 10.1371/journal.pone.0185066 (PMC5612686; doi:10.1371/journal.pone.0185066)
Supplement: S2 Table — (PDF) [file pone.0185066.s006.pdf]

S2 Table. Characteristics of participants and dropouts at baseline survey

		Participants N=5,676 n (%)	Dropouts[a] N=1,035 n (%)	p-value[b]
**Demographic factors**				
Age (years)				**<0.001**
	1	3,788 (67)	266 (26)	
	2	683 (12)	248 (24)	
	3	596 (11)	256 (25)	
	4	609 (11)	265 (26)	
Sex				0.321
	Female	2,733 (48)	481 (46)	
	Male	2,943 (52)	554 (54)	
Ethnicity				**<0.001**
	Whites	4,333 (76)	606 (59)	
	South Asian	1,343 (24)	429 (41)	
**Breastfeeding**				**0.002**
	Never	2,284 (40)	470 (45)	
	0-3 months	1,610 (28)	310 (30)	
	4-6 months	705 (12)	111 (11)	
	>6 months	1,077 (19)	144 (14)	
**Socioeconomic status**				
Townsend deprivation index[c]				**<0.001**
	Deprived (Index>0)	2,617 (46)	602 (58)	
Family education[d]				**0.035**
	High	2,426 (43)	397 (38)	
**Environmental exposures**				
Day care attendance		2,330 (41)	615 (59)	**<0.001**
Number of older siblings				0.282
	0	2,347 (41)	409 (40)	
	1 or 2	2,895 (51)	511 (49)	
	>=3	424 (7)	112 (11)	
Mother smoked during pregnancy		865 (15)	199 (19)	**0.001**
Mother smokes currently		1,115 (20)	220 (21)	0.232
Pet (dog/cat/bird) ownership		2,174 (38)	333 (32)	**<0.001**
**Parental history of atopy (asthma, eczema, or hay fever)**				
Maternal atopy		2,519 (44)	431 (42)	0.103
Paternal atopy		2,108 (37)	363 (35)	0.205
**Parental history of eczema**				
Maternal eczema		1,270 (22)	227 (22)	0.753
Paternal eczema		707 (12)	121 (12)	0.491

[a] Children who participated at the baseline survey in 1998 and had information on breastfeeding, but did not answer to eczema question in any survey.

[b] P-value of Chi-square test, to evaluate the differences between participants and dropouts.

[c] An area-based deprivation score; higher values indicate greater deprivation.

[d] Parents (either father or mother) who completed their full-time education older than age of 16.
