# Supplementary material for: Association between breastfeeding and eczema during childhood and adolescence: A cohort study
Source: PLoS One. 2017 Sep 25;12(9):e0185066. doi: 10.1371/journal.pone.0185066 (PMC5612686; doi:10.1371/journal.pone.0185066)
Supplement: S3 Table — (PDF) [file pone.0185066.s007.pdf]

S3 Table. Prevalence of current eczema at different age groups by breastfeeding status

Age groups (years)	Total				Breastfeeding (Yes)				Breastfeeding (No)				p[a]
	Number of participants (N)	Current eczema (yes) n	Current eczema (yes) % (95% CI)	Missing n (%)	Number of participants (N)	Current eczema (yes) n	Current eczema (yes) % (95% CI)	Missing n (%)	Number of participants (N)	Current eczema (yes) n	Current eczema (yes) % (95% CI)	Missing n (%)	
1	3,235	1,156	36 (34-37)	31 (1)	1,889	677	36 (34-38)	17 (1)	1,346	479	36 (33-38)	14 (1)	0.9059
2	2,319	814	35 (33-37)	121 (5)	1,427	508	36 (33-38)	76 (5)	892	306	34 (31-37)	45 (5)	0.4861
3-4	1,703	470	28 (26-30)	117 (7)	1,070	319	30 (27-33)	63 (6)	633	151	24 (21-27)	54 (9)	0.0187
5-6	2,562	688	27 (25-29)	134 (5)	1,627	442	27 (25-29)	79 (5)	935	246	26 (24-29)	55 (6)	0.7531
7-9	2,669	654	25 (23-26)	42 (2)	1,674	407	24 (22-26)	26 (2)	995	247	25 (22-28)	16 (2)	0.7599
10-13	1,945	352	18 (16-20)	24 (1)	1,259	228	18 (16-20)	14 (1)	684	124	18 (15-21)	10 (1)	0.9636
14-17	1,811	330	18 (17-20)	10 (1)	1,188	214	18 (16-20)	7 (1)	623	116	19 (16-22)	3 (0)	0.7190

[a] P-value of Chi-square test of independence to determine whether the association with current eczema between children who had been breastfed and those who had not been breastfed is significant.
