# Supplementary material for: Association between breastfeeding and eczema during childhood and adolescence: A cohort study
Source: PLoS One. 2017 Sep 25;12(9):e0185066. doi: 10.1371/journal.pone.0185066 (PMC5612686; doi:10.1371/journal.pone.0185066)
Supplement: S4 Table — (PDF) [file pone.0185066.s008.pdf]

S4 Table. Association between breastfeeding duration and prevalence of current eczema during childhood and adolescence---restricted to children with complete information on all the confounders

Overall association between breastfeeding and current eczema

	**Unadjusted model (N=5,456)**		**Adjusted[a] model (N=5,456)**	
Breastfeeding duration	OR (95% CI)	p-value	OR (95% CI)	p-value
No breastfeeding	1.00	-	1.00	-
0-3 months	1.04 (0.92-1.17)	0.564	1.02 (0.90-1.15)	0.802
4-6 months	1.02 (0.88-1.20)	0.770	0.97 (0.82-1.13)	0.668
>6 months	0.99 (0.86-1.13)	0.842	0.98 (0.85-1.14)	0.827

Data are presented as odds ratios (ORs) with their 95% confidence intervals (CIs) and associated p-values, both for unadjusted and adjusted GEE models.

The baseline group consisted of children who had not been breastfed.

[a] Adjusted for age, sex, ethnicity, family education, Townsend deprivation index, day care attendance, number of older siblings, pet ownership (dog, cat, or bird), pre- and postnatal maternal smoking and parental atopy (defined as paternal or maternal history of asthma, hay fever, or eczema).
