# Supplementary material for: Association between breastfeeding and eczema during childhood and adolescence: A cohort study
Source: PLoS One. 2017 Sep 25;12(9):e0185066. doi: 10.1371/journal.pone.0185066 (PMC5612686; doi:10.1371/journal.pone.0185066)
Supplement: S5 Table — (PDF) [file pone.0185066.s009.pdf]

S5 Table. Association between breastfeeding duration and current eczema at different age groups

Unadjusted logistic regression models

	**Age 1 n=3,204**		**Age 2 n=2,198**		**Age 3-4 n=1,586**		**Age 5-6 n=2,428**		**Age 7-9 n=2,627**		**Age 10-13 n=1,919**		**Age 14-17 n=1,801**	
**Breastfeeding**	OR (95% CI)	p-value	OR (95% CI)	p-value	OR (95% CI)	p-value	OR (95% CI)	p-value	OR (95% CI)	p-value	OR (95% CI)	p-value	OR (95% CI)	p-value
No breastfeeding	1.00	-	1.00	-	1.00	-	1.00	-	1.00	-	1.00	-	1.00	-
0-3 months	1.05 (0.88-1.26)	0.563	1.03 (0.83-1.28)	0.777	1.26 (0.96-1.66)	0.090	1.10 (0.89-1.37)	0.386	0.93 (0.74-1.16)	0.511	1.05 (0.79-1.40)	0.722	0.88 (0.65-1.20)	0.427
4-6 months	1.04 (0.83-1.32)	0.723	1.15 (0.87-1.51)	0.320	1.53 (1.10-2.13)	0.011	0.90 (0.67-1.20)	0.466	1.05 (0.80-1.38)	0.745	0.91 (0.62-1.33)	0.616	1.21 (0.84-1.74)	0.315
>6 months	0.92 (0.75-1.13)	0.430	1.06 (0.84-1.34)	0.634	1.24 (0.92-1.68)	0.158	1.02 (0.80-1.30)	0.876	0.98 (0.77-1.25)	0.890	0.97 (0.71-1.33)	0.850	0.92 (0.67-1.27)	0.630

Adjusted[a] logistic regression models

	**Age 1 n=3,100**		**Age 2 n=2,141**		**Age 3-4 n=1,547**		**Age 5-6 n=2,356**		**Age 7-9 n=2,530**		**Age 10-13 n=1,862**		**Age 14-17 n=1,739**	
**Breastfeeding**	OR (95% CI)	p-value	OR (95% CI)	p-value	OR (95% CI)	p-value	OR (95% CI)	p-value	OR (95% CI)	p-value	OR (95% CI)	p-value	OR (95% CI)	p-value
No breastfeeding	1.00	-	1.00	-	1.00	-	1.00	-	1.00	-	1.00	-	1.00	-
0-3 months	1.01 (0.83-1.22)	0.914	1.08 (0.86-1.37)	0.515	1.30 (0.97-1.74)	0.082	1.03 (0.82-1.30)	0.808	0.89 (0.70-1.13)	0.332	0.90 (0.67-1.23)	0.516	0.92 (0.66-1.28)	0.605
4-6 months	0.97 (0.75-1.25)	0.810	1.12 (0.83-1.51)	0.455	1.53 (1.07-2.19)	0.021	0.84 (0.62-1.14)	0.254	0.94 (0.70-1.26)	0.658	0.76 (0.51-1.13)	0.179	1.17 (0.79-1.73)	0.446
>6 months	0.92 (0.72-1.14)	0.384	1.12 (0.86-1.46)	0.396	1.23 (0.88-1.71)	0.226	0.95 (0.73-1.23)	0.704	0.93 (0.71-1.20)	0.568	0.86 (0.61-1.20)	0.370	0.92 (0.65-1.31)	0.646

Data are presented as odds ratios (ORs) with their 95% confidence intervals (CIs) and associated p-values for both unadjusted and adjusted logistic regression models.

The baseline group consisted of children who had not been breastfed.

[a] Adjusted for sex, ethnicity, Townsend deprivation index, family education, day care attendance, number of older siblings, pre- and post-natal maternal smoking, pet ownership (dog, cat, or bird), and parental atopy (defined as paternal or maternal history of asthma, hay fever, or eczema).
