# Supplementary material for: Association between breastfeeding and eczema during childhood and adolescence: A cohort study
Source: PLoS One. 2017 Sep 25;12(9):e0185066. doi: 10.1371/journal.pone.0185066 (PMC5612686; doi:10.1371/journal.pone.0185066)
Supplement: S6 Table — (PDF) [file pone.0185066.s010.pdf]

S6 Table. Association between breastfeeding duration and prevalence of current eczema during childhood and adolescence—modified by maternal atopy, sex, ethnicity and maternal smoking

Association between breastfeeding and current eczema modified by maternal atopy				
	**Mothers with history of atopy[a] (n=2,437)**		**Mothers without history of atopy[a] (n=3,019)**	
Breastfeeding duration	OR (95% CI)	p-value	OR (95% CI)	p-value
No breastfeeding	1.00	-	1.00	-
0-3 months	1.02 (0.86-1.22)	0.818	1.02 (0.85-1.22)	0.866
4-6 months	1.23 (0.99-1.54)	0.061	0.79 (0.62-1.00)	0.053
>6 months	1.14 (0.93-1.40)	0.193	0.91 (0.74-1.11)	0.338
**Association between breastfeeding and current eczema modified by sex**				
	**Girls[b] (n=2,630)**		**Boys[b] (n=2,826)**	
Breastfeeding duration	OR (95% CI)	p-value	OR (95% CI)	p-value
No breastfeeding	1.00	-	1.00	-
0-3 months	1.01 (0.84-1.21)	0.940	1.01 (0.85-1.21)	0.866
4-6 months	0.97 (0.78-1.22)	0.815	0.96 (0.77-1.20)	0.724
>6 months	1.06 (0.86-1.30)	0.604	0.92 (0.75-1.12)	0.405
**Association between breastfeeding and current eczema modified by ethnicity**				
	**Whites[c] (n=4,190)**		**South Asians[c] (n=1,266)**	
Breastfeeding duration	OR (95% CI)	p-value	OR (95% CI)	p-value
No breastfeeding	1.00	-	1.00	-
0-3 months	1.07 (0.93-1.24)	0.319	0.88 (0.66-1.17)	0.385
4-6 months	0.99 (0.83-1.19)	0.953	0.95 (0.65-1.37)	0.777
>6 months	1.00 (0.85-1.17)	0.973	0.95 (0.68-1.33)	0.755
**Association between breastfeeding and current eczema modified by maternal smoking**				
	**Mothers smoked[d] (n=1,168)**		**Mothers did not smoke[d] (n=4,288)**	
Breastfeeding duration	OR (95% CI)	p-value	OR (95% CI)	p-value
No breastfeeding	1.00	-	1.00	-
0-3 months	1.08 (0.83-1.40)	0.564	1.01 (0.88-1.17)	0.876
4-6 months	0.64 (0.41-1.01)	0.054	1.03 (0.86-1.22)	0.765
>6 months	0.76 (0.50-1.15)	0.192	1.03 (0.88-1.20)	0.719

Data are presented as odds ratios (ORs) with their 95% confidence intervals (CIs) and associated p-values for adjusted generalized estimating equations (GEE) models.
The baseline group consisted of children who had not been breastfed.
Mothers with history of atopy: maternal history of asthma, hay fever or eczema.
All models are adjusted for age, sex, ethnicity, family education, Townsend deprivation index, day care attendance, number of older siblings, pet ownership (dog, cat, or bird), pre- and postnatal maternal smoking and maternal atopy.
[a] Adjusted exceptionally for maternal atopy.
[b] Adjusted exceptionally for sex.
[c] Adjusted exceptionally for ethnicity.
[d] Adjusted exceptionally for pre- and postnatal maternal smoking.
