# Supplementary material for: Association between breastfeeding and eczema during childhood and adolescence: A cohort study
Source: PLoS One. 2017 Sep 25;12(9):e0185066. doi: 10.1371/journal.pone.0185066 (PMC5612686; doi:10.1371/journal.pone.0185066)
Supplement: S7 Table — (PDF) [file pone.0185066.s011.pdf]

S7 Table. Associations between breastfeeding duration and incident eczema at ages 2, 4, and 6 years--restricted to children with complete information on all the confounders

**At age 2 years (1999 survey)**				
	**Unadjusted (n=1,373)**		**Adjusted[a] (n=1,373)**	
Breastfeeding duration	OR (95% CI)	p-value	OR (95% CI)	p-value
No breastfeeding	1.00	-	1.00	-
0-3 months	1.04 (0.72-1.49)	0.848	1.14 (0.78-1.66)	0.487
4-6 months	1.14 (0.73-1.80)	0.562	1.16 (0.72-1.87)	0.542
>6 months	1.29 (0.89-1.87)	0.195	1.48 (0.99-2.21)	0.053
**At age 4 years (2001 survey)**				
	**Unadjusted (n=1,315)**		**Adjusted[a] (n=1,315)**	
Breastfeeding duration	OR (95% CI)	p-value	OR (95% CI)	p-value
No breastfeeding	1.00	-	1.00	-
0-3 months	0.99 (0.67-1.46)	0.962	1.01 (0.67-1.52)	0.947
4-6 months	1.30 (0.82-2.08)	0.267	1.29 (0.79-2.12)	0.305
>6 months	1.19 (0.79-1.81)	0.406	1.24 (0.80-1.93)	0.343
**At age 6 years (2003 survey)**				
	**Unadjusted (n=1,104)**		**Adjusted[a] (n=1,104)**	
Breastfeeding duration	OR (95% CI)	p-value	OR (95% CI)	p-value
No breastfeeding	1.00	-	1.00	-
0-3 months	0.93 (0.63-1.36)	0.702	0.96 (0.64-1.43)	0.826
4-6 months	0.80 (0.47-1.36)	0.404	0.85 (0.49-1.48)	0.568
>6 months	0.86 (0.56-1.33)	0.505	0.84 (0.53-1.31)	0.440

Data are presented as odds ratios (ORs) with their 95% confidence intervals (CIs) and associated p-values, both in unadjusted and adjusted logistic regression models.
The baseline group consisted of children who had not been breastfed.
[a] Adjusted for sex, ethnicity, family education, Townsend deprivation index, day care attendance, number of older siblings, pet ownership (dog, cat, or bird), pre- and postnatal maternal smoking, and parental atopy (defined as paternal or maternal history of asthma, hay fever, or eczema).
