# Supplementary material for: Association between breastfeeding and eczema during childhood and adolescence: A cohort study
Source: PLoS One. 2017 Sep 25;12(9):e0185066. doi: 10.1371/journal.pone.0185066 (PMC5612686; doi:10.1371/journal.pone.0185066)
Supplement: S8 Table — (PDF) [file pone.0185066.s012.pdf]

S8 Table. Association between breastfeeding duration and recurrent eczema

	Unadjusted (n=2,983)		Adjusted[a] (n=2,895)	
Breastfeeding	OR (95% CI)	p-value	OR (95% CI)	p-value
No breastfeeding	1.00	-	1.00	-
0-3 months	1.08 (0.89-1.30)	0.443	1.04 (0.84-1.27)	0.736
4-6 months	1.24 (0.97-1.58)	0.082	1.11 (0.85-1.45)	0.437
>6 months	1.07 (0.86-1.33)	0.531	0.98 (0.77-1.24)	0.857

Data are presented as odds ratios (ORs) with their 95% confidence intervals (CIs) and associated p-values for both unadjusted and adjusted logistic regression models.
The baseline group consisted of children who had not been breastfed.
[a] Adjusted for age, sex, ethnicity, Townsend deprivation index, family education, day care attendance, number of older siblings, pre- and post-natal maternal smoking, pet ownership, and parental atopy (defined as paternal or maternal history of asthma, hay fever, or eczema).
