# Supplementary material for: Association between breastfeeding and eczema during childhood and adolescence: A cohort study
Source: PLoS One. 2017 Sep 25;12(9):e0185066. doi: 10.1371/journal.pone.0185066 (PMC5612686; doi:10.1371/journal.pone.0185066)
Supplement: S9 Table — 3-month interval, 1-year-old children, n = 463. (PDF) [file pone.0185066.s013.pdf]

S9 Table. Level of agreement of responses for children with and without eczema who provided breastfeeding responses (yes/no) at baseline survey and in subsequent questionnaires

(3-month interval, 1-year-old children, n=463)

	++	+-	-+	--	Total	k
**Breastfeeding**						
Children with eczema at baseline	105	1	0	52	158	0.99
Children without eczema at baseline	192	5	6	102	305	0.92
**Total**					463	

++ positive on both baseline and repeated questionnaires

+- positive on baseline questionnaires only

-+ positive on repeated questionnaires only

-- negative on both baseline and repeated questionnaires

Level of agreement indicated by k: <=0.4 poor, 0.41–0.60 moderate, 0.61–0.80 good, >0.8 excellent
